# Strong Modulations of Optical Reflectance in Tapered Core–Shell Nanowires

**DOI:** 10.3390/ma12213572

**Published:** 2019-10-31

**Authors:** Francesco Floris, Lucia Fornasari, Vittorio Bellani, Andrea Marini, Francesco Banfi, Franco Marabelli, Fabio Beltram, Daniele Ercolani, Sergio Battiato, Lucia Sorba, Francesco Rossella

**Affiliations:** 1 Tyndall National Institute, University College Cork, T12 R5CP Cork, Ireland; 2 Dipartimento di Fisica, Università di Pavia and INFN, 27100 Pavia, Italy; 3 Department of Physical and Chemical Sciences, University of L’Aquila, 67100 L’Aquila, Italy; 4 Femto Nano Optics Group, Université de Lyon, CNRS, Université Claude Bernard Lyon 1, Institut Lumière Matière, F-69622 Villeurbanne, France; 5 NEST, Scuola Normale Superiore and Istituto Nanoscienze-CNR, 56127 Pisa, Italy

**Keywords:** core–shell, nanowires, semiconductors, optical reflectance, numerical simulations

## Abstract

Random assemblies of vertically aligned core–shell GaAs–AlGaAs nanowires displayed an optical response dominated by strong oscillations of the reflected light as a function of the incident angle. In particular, angle-resolved specular reflectance measurements showed the occurrence of periodic modulations in the polarization-resolved spectra of reflected light for a surprisingly wide range of incident angles. Numerical simulations allowed for identifying the geometrical features of the core–shell nanowires leading to the observed oscillatory effects in terms of core and shell thickness as well as the tapering of the nanostructure. The present results indicate that randomly displaced ensembles of nanoscale heterostructures made of III–V semiconductors can operate as optical metamirrors, with potential for sensing applications.

## 1. Introduction

Optical materials such as arrays of scattering building blocks with a subwavelength size and periodicity provide a well-established platform for light manipulation [1,2] together with extraordinary control over light–matter interaction at the nanoscale [3,4,5,6,7]. For instance, artificial calibrated arrangements of well-aligned dielectric nanostructures offer an ideal solution for engineering photonic bandgap materials and optoelectronic devices [8,9,10,11]. In this context, nanostructured optical reflectors have been proven to enable the control of polarization [12] and the orbital angular momentum of light [13], dispersive holograms [14], and spatial light modulators [15]. In addition, nanostructured composite systems theoretically allow for the design of metamaterials tailored for specific optical polarization properties, behaving as an almost perfect linearly dichroic system, quarter-wave plate, or circular polarizer [16]. Besides, the polarization-controlled manipulation of light has been demonstrated in the coupling of the whispering-gallery modes of a microresonator [17]. The quest for novel methods to realize functional assemblies of nanostructures characterized by conveniently tailored optical responses ceaselessly triggers the interest of scientists from many different areas: in this context, semiconductor nanowires (NWs) are emerging as a very promising class of quasi one-dimensional systems ideally suited for building up photonic platforms [18,19,20,21].

Attracted by the huge potential of NW-based systems for optical response engineering and light manipulation, in a previous work we studied the optical reflectance properties of nonperiodic assemblies of InAs NWs [22], probing their capability to manipulate electromagnetic waves (e.g., trapping, absorbing, enhancing, or bending a light beam) [23,24,25]. Polarization- and angle-resolved optical microreflectivity were investigated, and a modulation of the polarization-resolved reflectance as a function of the impinging photon energy and angle was observed. The experimental findings were reproduced in the whole wavelength range by numerical simulations, providing an experimental proof-of-concept that vertically aligned InAs NW arrangements represent a promising class of nanostructured optical reflecting surfaces. The observed features, unhampered by critical dependence on nanoscale parameters such as lattice constants, were quite robust against environmental fluctuations and thus are very promising for sensing and photovoltaic applications.

In this work, we extend our previous analysis by taking into account random assemblies of vertically aligned tapered core–shell (C–S) NWs. The usage of such radial heterostructures has been dictated by the will to amplify reflectance oscillations, acting only on NW morphology and not on their overall displacement, with the purpose of activating Fabry–Perot (F–P)-like resonances between the core and the shell of each scatterer, i.e., each NW. In particular, we investigated both experimentally and computationally the effect of marked geometrical anisotropies of tapered GaAs–AlGaAs C–S NW ensembles on the optical response in near- and far-field regimes. Angle-resolved specular reflectance measurements were performed on different samples, probing the dependence of the reflected light with respect to the impinging photon energy and angle. Finite-difference time-domain (FDTD) numerical simulations were carried out to support the comprehension of the collected spectra and to give an explanation in terms of electromagnetic field expansion. Occurrences of marked and robust resonances in the reflectance spectra are highlighted, as they precisely matched our simulation results. 

## 2. Materials and Methods 

**Figure 1:**
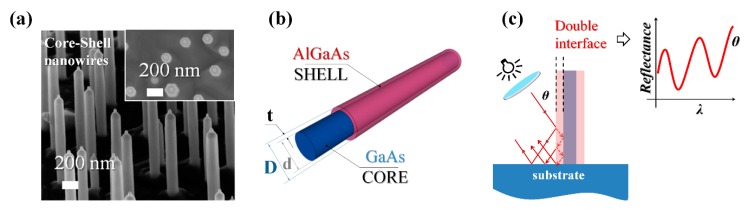
(**a**) 45° tilted scanning electron micrograph of a nontapered GaAs–AlGaAs core–shell nanowire sample qualitatively similar to sample B1 (a nanowire average length of 960 nm, a diameter of 150 nm, and a density of ~10 NW/µm^2^). Inset: at the top, the different contrasts along the radial direction indicate the different materials of the core and the shell; (**b**) Sketch of a core–shell nanowire (NW). The core and shell materials and the typical dimensions are indicated: an inner diameter of *d* ≈ 80 nm, an outer diameter of *D* ≈ 150 nm, and a shell thickness of *t* = 35 nm; (**c**) Schematics of the phenomena that occurred as light shined on the lateral surface of a core–shell NW: the latter behaved as a nanomirror, where the radial heterostructure implemented a double interface (air/shell and shell/core), likely yielding to marked interference effects.

**Figure 2:**
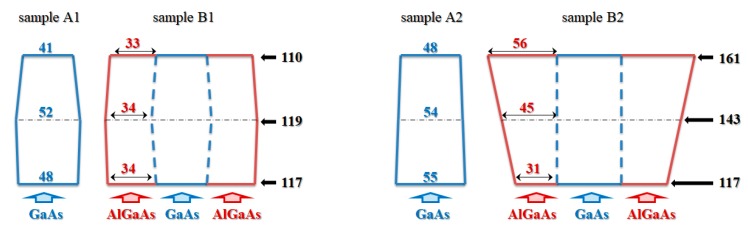
Schematic of the geometrical features of the four types of NW samples investigated in this work: not-tapered homogeneous GaAs NWs (samples A1 and A2), not-tapered core–shell GaAs–AlGaAs NWs (sample B1), and tapered core–shell GaAs–AlGaAs NWs (B2). Label numbers are expressed in nm and indicate: diameter of GaAs samples A1 and A2 (blue colored); thickness of AlGaAs shell in samples B1 and B2 (red colored); outer diameter of samples B1 and B2 (black colored).

GaAs NWs [26] and GaAs–AlGaAs core–shell NWs were grown on GaAs (111)B substrates (~500 µm thick) through gold-assisted chemical beam epitaxy [27]. In Figure 1, we report the scanning electron microscopy (SEM) micrographs of one of the samples grown for the present work (Figure 1a) together with a pictorial representation of an individual core–shell GaAs–AlGaAs NW (Figure 1b). The four samples grown for the present work were characterized using SEM (Supplementary Materials, Figure S1), and for each sample the morphology of at least 30 individual NWs was measured. This allowed us to identify the average outer (total) diameter, *D*, at the base, at the center, and at the tip of the NWs, as well as the average NW length. The standard deviation was used as uncertainty. The results of this morphological study are reported in Table 1. As we do in Table 1, in the next sections we will refer to the investigated samples as follows: Sample A1 is an ensemble of nontapered homogeneous GaAs NWs with an average outer diameter measured at the center of 52 ± 5 nm and an average length of 1535 ± 110 nm (grown at a temperature of *T* = 565 ± 10 °C). Sample B1 consists of nontapered core–shell GaAs–AlGaAs NWs with *D* = 119 ± 13 nm measured at the center of the NW, with an average AlGaAs shell thickness of 34 nm and an average length of 1274 ± 84 nm. The core and the shell of sample B1 were grown at the same growth temperature, *T* = 565 ± 10 °C, as sample A1. Sample A2 corresponds with nontapered homogeneous GaAs NWs with *D* = 54 ± 7 nm (measured at the center of NW) and an average length of 858 ± 99 nm (grown at *T* = 589 ± 10 °C). Finally, sample B2 consists of tapered core–shell GaAs–AlGaAs NWs, with *D* = 117 ± 12 nm at the NW base and *D* = 161 ± 14 nm at the NW tip and an average length of 1421 ± 66 nm. The core of sample B2 was grown at the same growth temperature as A2, while the AlGaAs shell was grown at Δ*T* = −100 °C. The difference in the morphology of samples B1 and B2 can be ascribed to the difference in the shell growth temperature. The geometrical feature characteristics of the four samples are represented in Figure 2. We noticed that both C–S samples were terminated with a thin GaAs capping layer to prevent oxidation of the AlGaAs shell. While the distribution of the NWs on the sample surface was random due to thermal dewetting of the Au particles [27], the four samples used in the experiments were prepared using the same protocol to achieve very similar NW densities, namely ~10 NW/µm^2^. Further details about the estimate of the NW density for each sample can be found in the Supplementary Materials, Figure S1).

Variable-angle specular reflectance was measured over a spectral range from 400 to 1200 nm. The light of a halogen lamp was collimated and then focused on the sample on a spot with an area of about 100 × 100 μm^2^ and a divergence less than 2°. A homemade microreflectometer setup associated with a Fourier-transform spectrophotometer (a IFS66, Bruker, Billerica, Massachusetts, MA, USA) was used to investigate the samples. For the dispersion measurements, a goniometer built in-house allowed for varying the value of the incidence angle *θ* between 10° and 70°, while the reflected beam was collected at 2*θ*. A Glan–Taylor polarizer was used to select transverse-electric (TE, or s-) or transverse-magnetic (TM, orp-) polarized light (electric or magnetic field perpendicular to the incidence plane, respectively). To measure the light reflected by the samples, a silicon photodiode was used. This variable-angle specular reflectance measurement setup, including the homemade microreflectometer and goniometer, was successfully exploited in previous works by some of us for an advanced characterization of the optical response of nanostructured materials, including not only semiconductors but also carbon nanotubes [28] and metallic nanostructures [29].

In order to explain and reproduce our experimental findings, numerical simulations were carried out using a model based on a random arrangement of vertically aligned identical NWs with diameters, lengths, and densities mimicking the investigated samples. As a starting point, we proved our assumptions, resorting to, for our computations, one of the most general numerical schemes for the solution of Maxwell equations in complex photonic media, i.e., the finite-difference time-domain (FDTD) method. The implemented numerical code was able to manage the full-wave nature of the electromagnetic field propagation and therefore to address the far- and near-field response of our structure, taking into account the electromagnetic (EM) field spatial distribution. In more detail, FDTD numerical simulations of the optical reflectance spectra and electric field distribution were performed with the commercially available Lumerical^®^ FDTD Solutions™ (version 8.18.1298 for x64, Lumerical Inc., Vancouver, BC, Canada) software [30]. We used the Lumerical^®^ material database to define the optical characteristics of both GaAs and AlGaAs, while SEM micrographs were used to evaluate the geometrical features of the NWs together with their density on the samples. The same code developed in our previous work [22] was used to manage the NW disposition: using the interpenetration of NWs placed on the substrate following the combination of five different Vogel spiral arrangements, we were able to satisfactorily mimic the randomness of our samples, and consequently, a specific section of the overall pattern was carefully selected to match the density requirements. An autogenerated nonuniform mesh of level six was found to be adequate after convergence tests, and periodic boundary conditions were chosen to recreate the entire nanostructured surface. Each simulation used to describe the sample response took two hours to run on a computer with a liquid-cooled 16-core processor (32 threads) and 64 GB of RAM. The electric field expansion was calculated for several horizontal *x*–*y* planes at different distances along the *z* axis (as defined in Figure 1c) from the upper surface of the substrate.

## 3. Results

**Figure 3:**
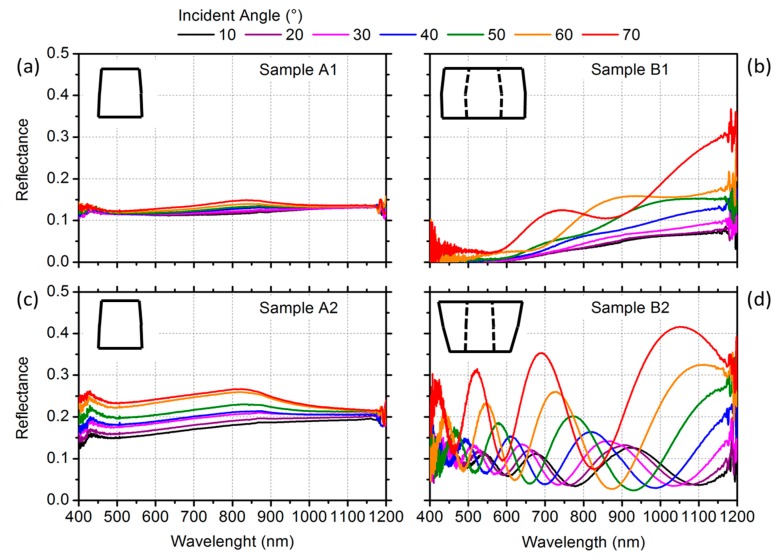
Reflectance experimental results for transverse-electric polarized incident light. The specular reflectance measured in (**a**,**c**) homogeneous GaAs NWs (samples A1 and A2); (**b**) nontapered GaAs–AlGaAs core–shell (C–S) NWs (sample B1); and (**d**) tapered GaAs–AlGaAs C–S NWs (sample B2).

In Figure 3, we report the angle-resolved specular reflectance (*R*) of homogenous GaAs NWs and core–shell GaAs–AlGaAs NWs characterized by different dimensions and morphology. *R* was measured for TE-polarized incident light covering the visible and near-infrared range as a function of the incident angle *θ*, which varied from 10° to 70°. Strong reflectance oscillations were immediately evident for the tapered radial heterostructures (panel d) with respect to the case of the nontapered one (panel b), and even more were evident with respect to the samples based on the homogeneous GaAs NWs (panels a and c). For the sake of completeness, a TM response was also collected, but no evidence of any relevant oscillating features was detected: this can be tentatively ascribed to the small scattering cross-section of the outer shell of a TM-polarized light beam.

In the assembly of homogenous GaAs NWs (Figure 3a,c) we observed that the reflectance spectra were mainly featureless, and only a weak shoulder at about 850 nm was (barely) evident. The shoulder is ascribable to the bulk GaAs response, and it slightly red-shifted as the incidence angle increased from 10° to 70°. The reflectance *R* showed a significantly different pattern for both GaAs–AlGaAs C–S NWs (Figure 3b,d). For small incident angles, the mean signal amplitude was, on average, lower with respect to the equivalent homogenous sample. The nontapered sample showed an overall decreasing trend by decreasing the wavelength, yielding an almost null value of *R* around 500 nm. When the incident angle was augmented, an *R* oscillation appeared. We ascribe the oscillations to interference effects rising from the presence of a double interface, namely the air–shell interface and the core–shell one. Extremely marked oscillations occurred in the reflectance spectra of the tapered sample (Figure 3d), which were very evident for every incidence angle. It is worthwhile noting that in this sample, *R* had a nonvanishing value at low wavelengths as well, and up to 40% of the incident light could be reflected. The extent and energy positions of peaks and dips evolved monotonically upon changes in the incidence angle.

**Figure 4:**
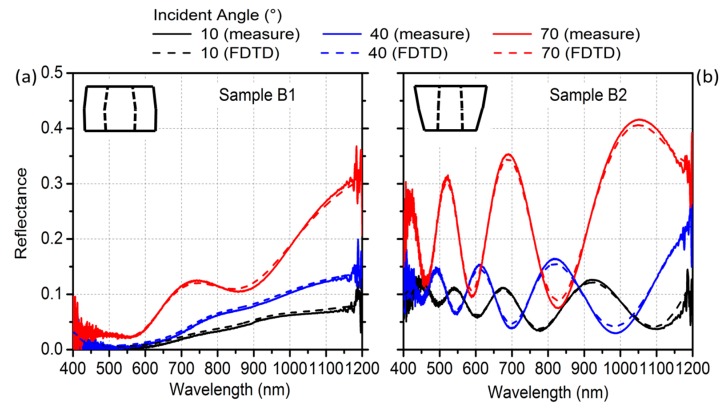
Comparison between experimental reflectance and finite-difference time-domain (FDTD) simulations. (**a**) Measured (solid line) and simulated (dashed line) specular reflectance for the nontapered C–S, sample B1; (**b**) measured (solid line) and simulated (dashed line) specular reflectance for the tapered C–S, sample B2.

In Figure 4, we report *R* calculated for the nontapered and tapered C–S NWs for three different incident angles *θ* (namely 10°, 40°, and 70°), as well as the corresponding experimental spectra: the agreement was evidently outstanding. In the specific case of the tapered C–S system, which experimentally allowed for achieving marked oscillations at every angle, we assumed that the tapered morphology promoted interference effects on the light scattered by the double surface of every NW. Since (i) the single NW geometrical dimensions were smaller or comparable to the wavelength of the incident light and (ii) the interdistance between adjacent NWs was in the order of the wavelengths, the far-field response of our systems was best addressed with a complete solution of the scattering processes, evaluating the diffraction pattern arising from the generalized Snell law [31].

**Figure 5:**
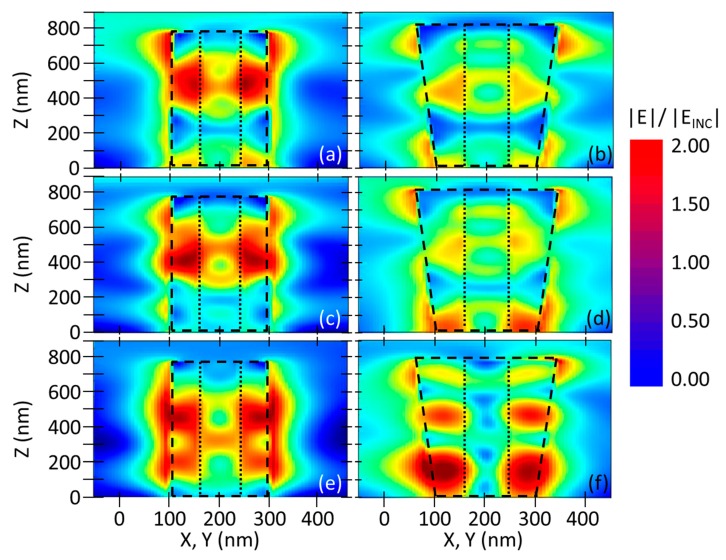
Longitudinal electric field intensity distribution around a NW with respect to the angle of incidence *θ*. Near-field normalized electric field expansion for the nontapered C–S geometry, (**a**) at an incident angle *θ* = 0° and a wavelength of 925 nm; (**c**) at an incident angle *θ* = 40° and a wavelength of 800 nm and (**e**) at an incident angle *θ* = 70° and a wavelength of 735 nm. Near-field normalized electric field expansion for the tapered C–S geometry, (**b**) at an incident angle of *θ* = 0° and a wavelength of 925 nm, (**d**) at an incident angle of *θ* = 40° and a wavelength of 820 nm and (**f**) at an incident angle *θ* = 70° and a wavelength of 690 nm.

In order to rationalize the experimental findings and rigorously substantiate our ansatz, we investigated the electric field (EF) spatial distribution around the NWs, as reported in Figure 5. The EF was mainly thickened in the shell layer and at the shell–air interface, and thus the corresponding expansion displayed maxima and minima around these two portions of the scatterers. In addition, the EF spot shifted consistently along the NW toward the substrate (lower *z* values) by increasing the incidence angle *θ* both for the untampered and tapered samples. The spot shape and localization, however, strongly differed for the nontapered (Figure 5a,c,e) and tapered (Figure 5b,d,f) C–S NWs. For the nontapered heterostructure, the EF remained confined in the proximity of the scatterers without any substantial intra-NW or substrate–NW interaction. Regarding the tapered C–S heterostructures, a quite important fraction of the EF was instead spread outside the scatterers, potentially enabling intra-NW coupling, and, for the higher incidence angle of *θ* = 70°, a further substrate–NW interaction could be established thanks to the tapered shape that reduced the angle between the air–shell external interface of the NWs and the substrate upper surface.

**Figure 6:**
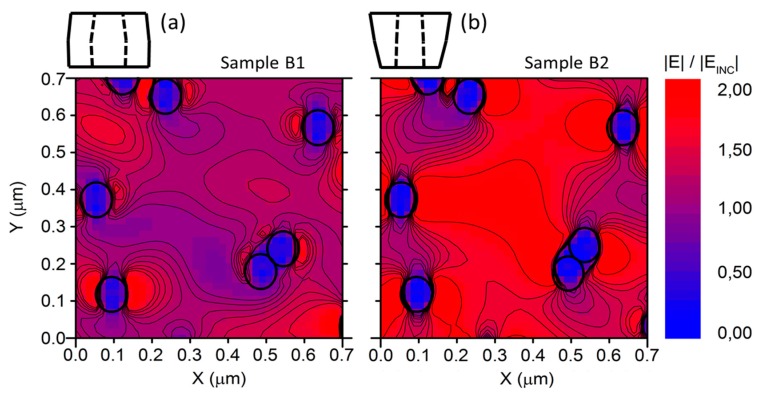
Transversal electric field intensity distribution on an *x*–*y* plane for *z* = 200 nm at an incident angle *θ* = 70°. Near-field normalized electric field expansion spatial distribution for (**a**) the nontapered C–S sample at a wavelength of 735 nm and (**b**) the tapered C–S sample at a wavelength of 690 nm. The black circles roughly indicate the core–shell interface of the NWs. *E_INC_* represents the incident electric field.

Calibrating the NW densities, we obtained a disordered series of localized resonators that were roughly circularly shaped and close enough to establish reciprocal coupling, as proven by the horizontal EF expansion shown in Figure 6. In this way, the impinging electromagnetic field was then mostly confined in the volume around the NWs and may have been rationalized as a combination of localized oscillations around the NWs coupled with traveling waves among the NWs themselves. This is crucial, since it shows that, at particular angles, the light reflected from the nanostructured surface could display marked broadband oscillations and provide a predictive tool to engineer them. In general, the near-field distribution was qualitatively almost identical for the nontapered and tapered cases, but the normalized intensity was stronger for wavelengths that gave constructive interference effects. 

## 4. Discussion

Generally, the electromagnetic (EM) response of an assembly of nanostructures is dictated by the superposition of the individual scatterer responses and by the scatterer spatial distribution. As for the latter, while in ordered arrays the response to an impinging EM field is best expressed in the form of a plane-wave multiplied by a periodic function (Bloch function), in our case the random arrangement of the NWs forbade the applicability of the Bloch theorem. Notwithstanding the lack of a lattice superstructure dominating the electromagnetic response, we could shape the EM field distribution, addressing the individual scatterer response. As a matter of fact, the oscillating behavior in the optical response of our sample was achieved by acting on the NW morphology: we resorted to a multilayered radial structure to implement a multiple refractive index contrast, and we tailored the nanostructure profile along the longitudinal direction to introduce a preferential directionality of the reflected light toward the substrate. The combination of these two aspects activated and boosted the reported interference effects that, sustained by the entire ensemble of NWs, gave rise to exceptionally large oscillations in the reflectance. Our scatterers can likely be regarded as distributed Bragg refractors with cylindrical symmetry, exploiting the refractive index contrast between three different media, i.e., the air, the AlGaAs shell, and the GaAs core. Consistently, the reported phenomenology was similar to the one usually observed in planar layer-by-layer systems and can be accounted for in terms of wave propagation in cylindrical symmetry geometry. 

Noticeably, the distributed EM network showed by the FDTD simulations, which was correlated with the NW average density but independent of the specific surface arrangement, could provide enhanced sensitivity to changes in the surrounding refractive index and a consequent pronounced shift of the interference fringes, making these assemblies of semiconductor nanostructures very promising and robust for sensing applications. Sharing the potential for and perspectives on sensing applications with similar systems of disordered semiconductor nanowires [22], the present system allows for controlling additional degrees of freedom, namely the radial heterostructure and tapering, that can be exploited to “fine-tune” the process of engineering an optical response. In more detail, the potential interest in the results presented in this work for the development of innovative sensors is manifold. First of all, the large surface-to-volume ratio of NWs and NW-based systems have great potential for sensing applications at large, as has been demonstrated in the exploitation of different platforms and approaches and in envisioning electronic transport experiments as well as an all-optical paradigm. On the one hand, semiconductor nanowire field-effect sensor devices have been proposed as detection systems for biological or chemical species in solution [32], while conductometric sensors developed to exploit single InAs NWs have been recently demonstrated [33]. On the other hand, all-optical sensing schemes compatible with microfluidic technologies have been recently proposed for the case of InAs NWs [22]. In this context, the optical response of the NW assembly can be analytically estimated for different filling media (i.e., different refractive indexes in the space surrounding the NWs) in the frame of an effective model [34,35], and the fact that the reflectance modulations reported in our samples were not strictly dependent on the geometry of the arrangement yielded a quite robust and stable optical response and protected it from fluctuations of the NW-to-NW distance. Following this approach, all-optical NW-based sensing platforms compatible with microfluidic integration [36,37] can be envisioned, where the sensing area of the chip is made of NWs, light is focused at a fixed incidence angle, and the reflectance versus wavelength is probed. Specifically, a small quantity of fluid has to be driven within a microfluidic circuit into the NW-based reflector, so that when the liquid fills the gaps around the NWs, a net change of the reflectance spectrum is observed. This platform can be used to measure the refractive index of the liquid by comparing the experimental curve against a calibration library, or, more interestingly, it could enable the detection of a specific biological complex formation [38,39] upon the functionalization of NW surfaces with biorecognition elements.

Finally, in support of the experimental as well as theoretical findings reported and discussed above, we also carried out additional simulations in order to widen the parameter space covered by our investigation. In particular, we explored the following four cases: (i) NWs with the same geometrical shapes and material compositions of the measured ones, but with a total length that was half of the actual investigated structures; (ii) homogeneous GaAs NWs with C–S geometry; (iii) C–S NWs with half of the radius of the shell; and (iv) C–S NWs with a doubled radius of the core. The results of these simulations, which are reported in detail in the Supplementary Materials, Figure S2–S5, confirmed that the main underlying mechanism of the observed reflectance oscillations was the coexistence of two key features, namely (1) the Fabry–Pérot interference effects arising in the properly tuned volume of the shell of every NW coupled with (2) the interference effects established between the external surface of the NWs and the substrate, which were promoted by a tapered shape that gave rise to a favorable angle so that the light was reflected multiple times in the volume between the NWs, creating a net enhancement of the oscillation intensity.

## 5. Conclusions

In conclusion, we exploited bottom-up semiconductor nanowire technology to demonstrate a novel class of optical materials based on random assemblies of vertically aligned tapered core–shell GaAs–AlGaAs nanowires. Our assemblies of semiconductor nanostructures acted as optical reflectors, where the intensity of the reflectance could be engineered starting from a suitable choice of materials, average nanostructure dimensions, and morphology. We highlight that the response of our system was mainly driven by the refractive index contrast occurring in the materials. This was particularly evident in the case of core–shell NWs, where the presence of the two interfaces together with a proper tailored shape promoted interference phenomena, yielding to exceptionally marked and robust broadband oscillations.

## Figures and Tables

**Table 1:** Morphological parameters of the investigated samples.

Sample	Average Length (nm)	Average Outer Diameter (nm)		
-	-	Base	Center	Tip
A1	1535	48	52	41
B1	1274	117	119	110
A2	858	55	54	48
B2	1421	117	143	161

## Supplementary Materials

The following are available online at https://www.mdpi.com/1996-1944/12/21/3572/s1, Figure S1. SEM micrographs of the four NW samples investigated in this work, Figure S2. Simulated specular reflectance results for a 40 degree TE polarized incident light, Figure S3. Simulated specular reflectance results for a 40 degree TE polarized incident light, Figure S4. Simulated specular reflectance results for a 40 degree TE polarized incident light, Figure S5. Simulated specular reflectance results for a 40 degree TE polarized incident light.
