# Supplementary material for: Strong Modulations of Optical Reflectance in Tapered Core–Shell Nanowires
Source: Materials (Basel). 2019 Oct 31;12(21):3572. doi: 10.3390/ma12213572 (PMC6862277; doi:10.3390/ma12213572)
Supplement: Supplementary file 1 [file materials-12-03572-s001.pdf]

materials

*Article*

# Strong Modulations of Optical Reflectance in Tapered Core–Shell Nanowires

**Francesco Floris [1,*], Lucia Fornasari [2], Vittorio Bellani [2], Andrea Marini [3], Francesco Banfi [4], Franco Marabelli [2], Fabio Beltram [5], Daniele Ercolani [5], Sergio Battiato [5], Lucia Sorba [5] and Francesco Rossella [5,*]**

[1] Tyndall National Institute, University College Cork, Cork T12 R5CP, Ireland; francesco.floris@tyndall.ie
[2] Dipartimento di Fisica, Università di Pavia and INFN, Pavia 27100, Italy
[3] Department of Physical and Chemical Sciences, University of L'Aquila, L'Aquila 67100, Italy
[4] Femto Nano Optics Group, Université de Lyon, CNRS, Université Claude Bernard Lyon 1, Institut Lumière Matière, F-69622 Villeurbanne, France
[5] NEST, Scuola Normale Superiore and Istituto Nanoscienze-CNR, Pisa 56127, Italy
* Correspondence: francesco.floris@tyndall.ie (F.F.); francesco.rossella@sns.it (F.R.)

## 1. SEM Study of the Investigated NW Samples

The four investigated samples have been preliminary observed with SEM to assess their main morphological features. In Figure S1, SEM micrographs at increasing magnifications and for different tilting are report for each NW samples. By analyzing the top-view SEM micrographs (Figure S1, right column) we estimated the average NW density (average number of NW per $um^2$) for each of the four investigated samples. In particular we estimated the number of NWs on a growth substrate area of 1 $um^2$ over 20 different areas for each samples. We notice that our samples are quite homogeneous in terms of the distribution of the areal density of NWs. The average NW density for each sample is reported in the following: Sample A1 (NW1765_Ga): 8 NWs/$um^2$; Sample B1 (NW1802_GaAlGa): 8 NWs/$um^2$; Sample A2 (NW1717_Ga): 13 NWs/$um^2$; Sample B2 (NW1720_GaAlGa): 9 NWs/$um^2$.

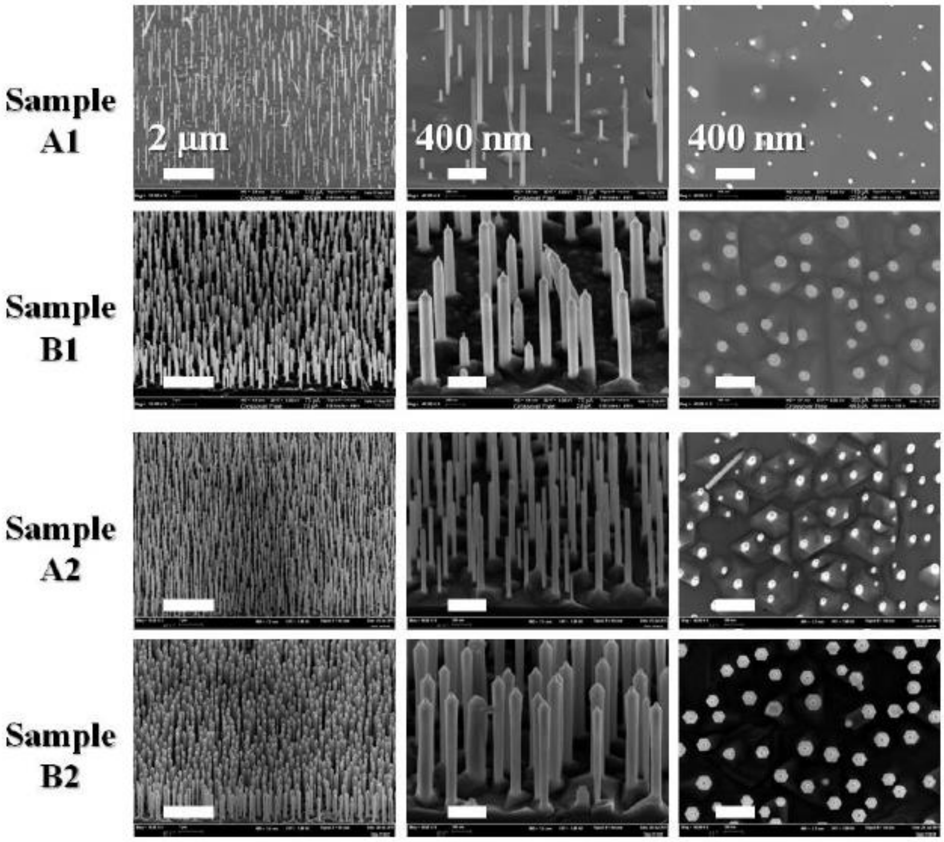


**Figure S1.** SEM micrographs of the four NW samples investigated in this work, namely sample A1 and B1, and sample A2 and B2 (see main text for details). The left- and center-column report 45° tilted SEM micrographs, while the right column display top-view SEM micrographs of the different samples. Scale bars and labels indicated for sample A1 are constant along each column.

## 2. Investigating the Parameter Space: Additional FDTD Studies

Further FDTD numerical simulations were carried out to increase the confidence in the understanding of the collected and simulated spectra. Four cases were taken into account: NWs with the same geometrical shapes and material compositions of the measured ones but with a total length that is half with respect to the actual investigated structures (Figure S2), homogeneous GaAs NWs with the C-S geometries (Figure S3), C-S NWs with half of the radius of the shell (Figure S4) and C-S NWs with a doubled radius of the core (Figure S5).

In the first case (Figure S2), we simulated the reflectance response of NWs with the same geometrical shapes and material compositions of the measured ones but with a total length that is half with respect to the actual investigated structures. The longitudinal dimension of the NWs is then smaller and this means a smaller surface able to reflect the incident light. In the homogeneous GaAs NWs, this produces just a small reduction of the signal intensity that can be explained by the reduction of the material that is reflecting the incident flight; in the GaAs-AlGaAs C-S NWs, the effect of the reduction of the material also corresponds to a decrease in the volume available for the establishment of the Fabry–Pérot effects with a consequent drastic suppression of the interference effects on the light scattered by the double-surface of every NW. The overall result is a decreasing of the reflectance signal with a consequential reduction of the oscillatory effect and a flattening of the average behavior.

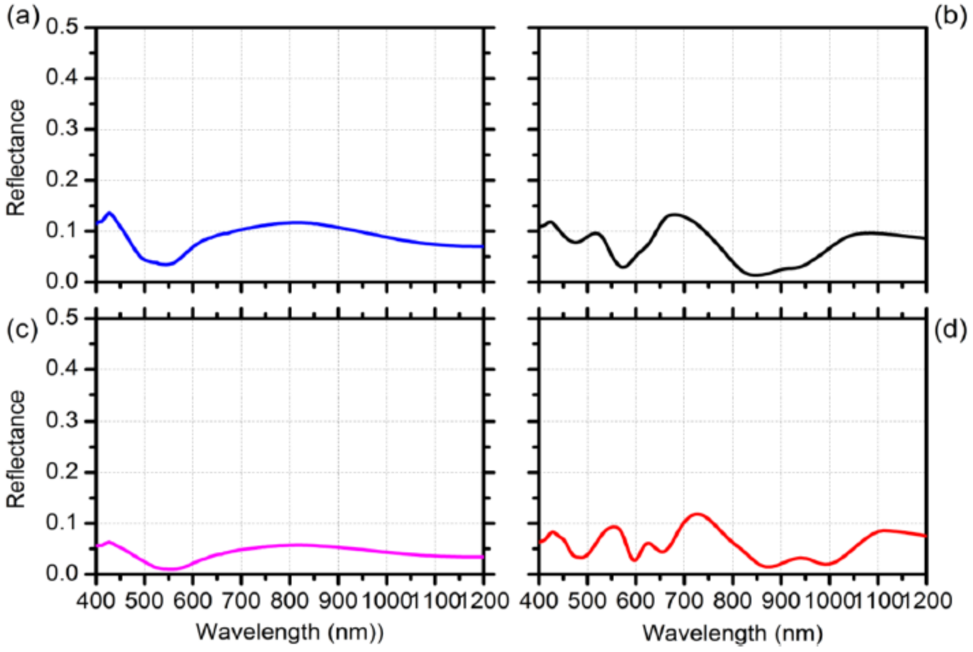


**Figure S2.** Simulated specular reflectance results for a 40 degree TE polarized incident light. Simulated specular reflectance of NWs with a total length that is half with respect to the measured ones: (**a,c**) homogeneous GaAs NWs, deriving from samples A1 and A2 respectively, (**b**) non-tapered GaAs-AlGaAs C-S NWs, deriving from sample B1, and (**d**) tapered GaAs-AlGaAs C-S NWs, deriving from sample B2.

In the second case (Figure S3), we simulated the reflectance response of NWs with the C-S geometries but a homogeneous GaAs material composition. The total absence of the core-shell interface corresponds to the absence of any volume available for the establishment of the Fabry–Pérot effects and the overall result is, again, a decreasing of the reflectance signal and a flattening of the average behavior. No mechanism is now active in establishing proper oscillations and, consequently, no gain effect is detected. Only a minor interference effect is present in the tapered homogeneous GaAs NWs with the geometry of sample B2, panel b, reasonably arising from an interference effect activated by the tapered shape of the NWs that helps the field to slightly interact with the substrate.

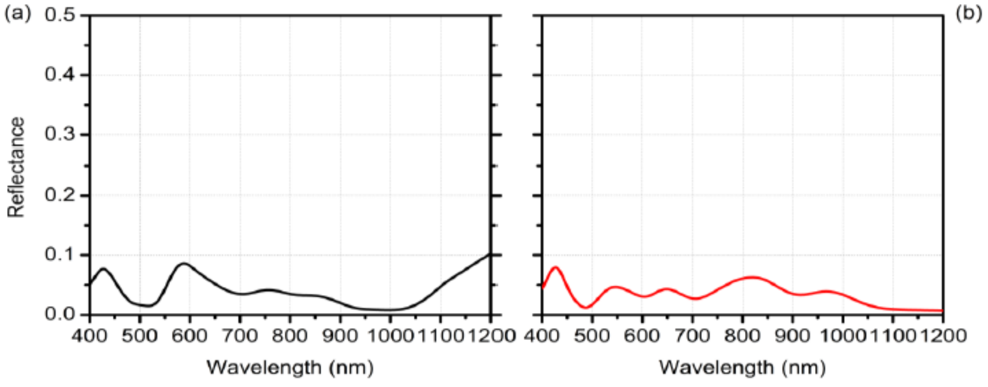


**Figure S3.** Simulated specular reflectance results for a 40 degree TE polarized incident light. Simulated specular reflectance of homogeneous GaAs NWs with the C-S geometries: (**a**) non-tapered homogeneous GaAs NWs with the geometry of sample B1, and (**b**) tapered homogeneous GaAs NWs with the geometry of sample B2.

In the third and fourth cases (Figure S4 and S5 respectively), we simulated the reflectance response of GaAs-AlGaAs C-S NWs with half of the radius of the shell (the radius of the core was

kept unchanged) in case three (Figure S4) and C-S NWs with a doubled radius of the core (the radius of the shell was kept unchanged) in case four (Figure S5). From the practical point of view, we detuned the Fabry–Pérot cavity, changing the shell thickness and, reasonably, the effects is similar in both cases: a complete suppression of the interference effects on the light scattered by the double-surface of every NW.

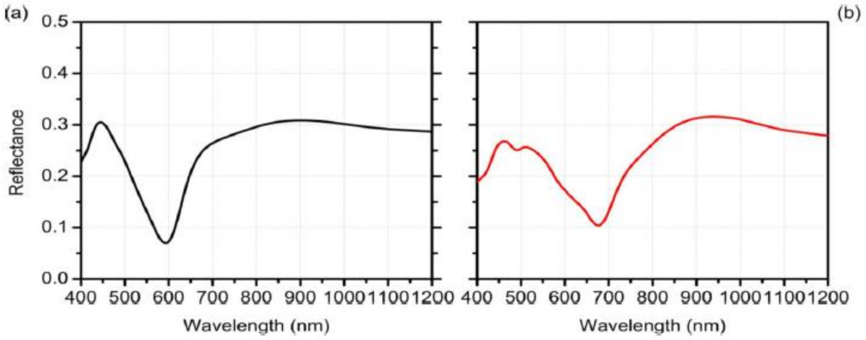


**Figure S4.** Simulated specular reflectance results for a 40 degree TE polarized incident light. Simulated specular reflectance of: (**a**) non-tapered GaAs-AlGaAs C-S NWs with the same core radius and half of the radius of the shell of sample B1, and (**b**) tapered GaAs-AlGaAs C-S NWs with the same core radius and half of the radius of the shell of sample B2.

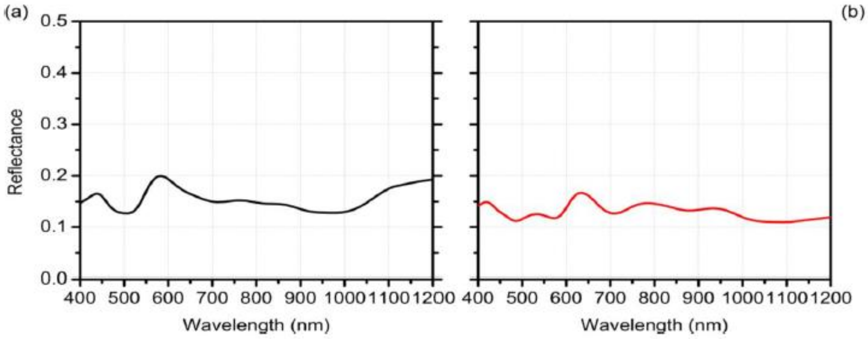


**Figure S5.** Simulated specular reflectance results for a 40 degree TE polarized incident light. Simulated specular reflectance of: (**a**) non-tapered GaAs-AlGaAs C-S NWs with the same shell radius but radius of the core that is double with respect to sample B1 and (**b**) tapered GaAs-AlGaAs C-S NWs with the same shell radius but radius of the core that is double with respect to sample B2.

© 2019 by the authors. Submitted for possible open access publication under the terms and conditions of the Creative Commons Attribution (CC BY) license (http://creativecommons.org/licenses/by/4.0/).
